# Visual and Refractive Outcomes of Toric Implantable Phakic Contact Lens in Stable Keratoconus - A retrospective interventional cross-sectional study

**DOI:** 10.22336/rjo.2025.54

**Published:** 2025

**Authors:** Kodavoor Shreesha Kumar, Raju Sumithra, Pandey Ujjwala, Dandapani Ramamurthy, Ramamurthy Chitra, Ramamurthy Shreyas, Sachdev Gitansha

**Affiliations:** 1 Department of Cornea Services, The Eye Foundation, Coimbatore, Tamil Nadu, India; 2 Department of Cornea, Cataract and Refractive Surgery, The Eye Foundation, Coimbatore, Tamil Nadu, India; 3 Department of Cataract, The Eye Foundation, Coimbatore, Tamil Nadu, India; 4 Department of Cataract and Glaucoma Services, The Eye Foundation, Coimbatore, Tamil Nadu, India

**Keywords:** Implantable Toric Phakic Contact Lens, keratoconus, collagen cross-linking, topography-guided photorefractive keratectomy, intracorneal ring segments, IOL = Intraocular lens, TICL - Toric implantable Collamer lens, TIPCL = Toric implantable phakic contact lens, PTK = Phototherapeutic keratectomy, PRK = Photorefractive Keratectomy, ICRS = Intracorneal ring segments, CXL = Collagen cross linking, UDVA = Uncorrected distance visual acuity, BDVA = Best corrected distance visual acuity, ACD = Anterior Chamber Depth, W-W = white to white, SE = spherical equivalent, LogMAR = Logarithm of the minimal angle of resolution, D = dioptre, OVD = Ophthalmic viscoelastic devices, PIOL = Phakic intraocular lens

## Abstract

**Objective:**

Refractive management in keratoconus is a challenging task. The study aimed to evaluate the results of Toric Implantable Phakic Contact Lenses in providing visual rehabilitation for patients with stable keratoconus after one year of primary procedures, including collagen cross-linking, topo-guided photorefractive keratectomy, and intracorneal ring segments.

**Methods:**

This retrospective cross-sectional interventional study was conducted at a tertiary eye care hospital in South India. Sixty-four patients with 86 eyes were included, and all of them received Toric Implantable Phakic Contact Lens (TIPCL) implantation. All patients were subsequently divided into three subgroups: collagen cross-linking (CXL), intracorneal ring segments (INTACS+CXL), and Topographically Guided photorefractive keratectomy (TGPRK+CXL). Patients were followed up regularly for a period of one year.

**Results:**

We included data from 64 patients with 86 eyes, with an average age of 30.67 ± 7.96 years. Reduction in the mean spherical equivalent, sphere, and cylinder values was observed across all three subgroups, and the results were statistically significant. There was considerable improvement in uncorrected distance visual acuity (UDVA) and best-corrected distance visual acuity (BDVA). At the end of one year, 75.3% of eyes achieved UDVA improvement of more than 3 lines. The average safety index was 0.77, and the efficacy index was 1.20.

**Discussion:**

TIPCL implantation proved to be a safe and effective option for correcting residual refractive error in keratoconus patients after stabilization procedures. Our results align with previous reports on phakic IOLs, demonstrating comparable safety and efficacy indices. The study emphasizes the importance of careful patient selection, as outcomes are strongly influenced by baseline refraction, corneal irregularity, and disease stability. While complications such as lens rotation, shallow anterior chamber, and cataract were rare, they emphasize the need for meticulous surgical planning and postoperative monitoring. Compared with existing literature, this is among the first studies to analyze outcomes across three stabilization subgroups, underscoring TIPCL as a viable, reversible option for patients with stable keratoconus.

**Conclusion:**

The implantation of Toric Implantable Phakic Contact Lens (TIPCL) is a valuable option for visual rehabilitation in keratoconus patients.

## Introduction

Keratoconus is a common primary corneal ectatic disorder, generally presenting bilaterally and often asymmetrical. It is characterized by localized corneal thinning and bulging. Factors such as male sex, eye rubbing, a family history of the condition, and related conditions like asthma, allergies, and eczema are recognized as risk factors for keratoconus [1,2]. Abnormal corneal curvature resulting from structural changes in stromal collagen can lead to progressive myopia, irregular astigmatism, and scarring, ultimately resulting in poor visual acuity. Collagen cross-linking (CXL) is a proven effective procedure to halt the progression of disease. Still, sequential vision restorative options are required for addressing the irregular astigmatism and residual refractive error [3]. The management of keratoconus varies according to its severity to achieve the best corrected visual acuity. Mild and stable keratoconus in its early phase is usually treated with corrective spectacles. But as the disease progresses to moderate or severe forms, achieving satisfactory vision becomes more difficult. In these cases, specialized contact lenses are often used to enhance vision, although not all patients find them comfortable. Options like topo-guided photorefractive keratectomy (TGPRK), intracorneal ring segments (ICRS), phakic intraocular lenses (PIOL), and corneal transplants offer potential solutions [4-7]. Lamellar keratoplasty, also known as penetrating keratoplasty, was considered a last resort option for patients with advanced keratoconus and corneal scarring due to its associated complications [8].

Phakic intraocular lenses (pIOLs) are an effective refractive solution for keratoconus, as they address the full refractive error without modifying the corneal surface or image size, and they can be removed if refractive changes occur due to disease progression [9].

Most studies on the effectiveness of phakic intraocular lenses in patients with keratoconus have primarily focused on the implantable contact lens (ICL). However, another type of posterior chamber phakic lens, known as the implantable phakic contact lens (IPCL), produced by Care Group in India, has also been studied for its efficacy and safety [10].

This study aims to compare the efficacy and safety of IPCL over one year in patients with keratoconus, as no similar studies have been reported in the literature to date.

## Materials and methods

This retrospective, cross-sectional, interventional study was conducted at a tertiary eye care hospital in Southern India. The protocol was registered and approved by the Ethics Committee of our Institute and adhered to the principles outlined in the Declaration of Helsinki. A total of 86 eyes were included in this retrospective interventional study, all of which had undergone TIPCL implantation following 12 months of CXL or CXL plus procedure (CXL combined with INTACS and TGPRK) between 2017 and 2022.

All 64 patients were subdivided into three groups: CXL, INTACS+CXL, and TGPRK+CXL. Inclusion criteria were as follows: participants older than 21, best-corrected distance visual acuity of 20/40 or better in treating eye, stable refraction (change in mean refractive spherical equivalent of not more than 0.25 D) for a minimum period of 1 year, intraocular pressure (IOP) less than 21 mmHg, endothelial count of >2,200 cells/mm^2^, anterior chamber depth (ACD) ≥ 2.8 mm and k max of less than 55 D. Patients were considered eligible for implantation of IPCL following CXL only when the keratoconus condition was considered stable. Stability was defined as subjective refractions within ±0.50 dioptres of the spherical equivalent at one year, and this stability was typically consistent with the refraction recorded before the Toric IPCL procedure.

The conditions that led to exclusion from the study were: clinically significant irregular astigmatism, ACD (anterior chamber depth) <2.8 mm, progressive keratoconus with poor spectacle visual acuity, advanced keratoconus with scarring, previous herpetic keratitis, active inflammatory ocular disease, cataract, glaucoma, any form of retinal disorder, pregnant and lactating females, and autoimmune disorders.

Preoperatively, the following parameters were considered: logarithm of the minimal angle of resolution (logMAR) of UDVA, logMAR of BDVA, IOP using Goldmann applanation tonometry, manifest and cycloplegic refraction, evaluation of the anterior and posterior segments with slit-lamp microscopy, retinal examination via direct and indirect ophthalmoscopy, and gonioscopy for peripheral angle analysis. Scheimpflug corneal topography (Pentacam HR; Oculus Optikgerate GmbH, Wetzlar, Germany) was used to assess keratometric readings, anterior and posterior corneal curvature, corneal thickness, and endothelial cell count by specular microscopy (Tomey EM-3000, Tomey Co.), which were documented for each patient. Axial length, anterior chamber depth, pupil size, and white-to-white diameter (W-W) were measured using a laser interferometry biometer (Lenstar, Haag Streit, USA) and handheld digital calipers. Anterior chamber depth and W to W (distance from the corneal endothelium to the anterior lens capsule) measurements were taken to select the correct size phakic IOL.

The IPCL V 2.0 Toric (Caregroup Sight Solution Private Limited, Vadodara, Gujarat, India) is composed of hydrophilic acrylic, a piece, and posterior chamber phakic IOL (PIOL). It is foldable, injectable, and designed to be implanted behind the iris with the haptic zone resting on the ciliary sulcus. It features six haptics and two holes in the upper peripheral zone, four holes outside the optical zone, and an additional central hole (380 micrometers) to facilitate aqueous humor outflow, thereby eliminating the need for prior iridotomy. Its dioptric power range is designed to correct myopia from -1.00 to -30.00 Diopters (D) and astigmatism (up to -10D in 0.5D increments). It was developed with an aspheric optic zone with zero aberration. The diameter range of the optic is from 5.75 to 6.20 mm and 11.0 mm to 14.00 mm overall diameter, and it can be customized for large scotopic pupil (6.5/6.8/7.2/7.5) and high astigmatism (>10D).

### Surgical technique

Before intraocular intervention, written and informed consent was received from all subjects after a brief explanation of the anticipated visual outcomes of the procedure. The manufacturer selected the lens power for each patient. All patients underwent the procedure by a single senior phaco-refractive surgeon using topical anaesthesia (proparacaine hydrochloride ophthalmic solution 0.5%). Eyes were dilated with tropicamide and phenylephrine. After cleaning and draping, a self-sealing temporal clear corneal wound was created using a 3 mm disposable keratome. Two side ports were made with an MVR (microvitreoretinal) blade. Low-molecular-viscoelastic devices (APPAVISC, Hydroxypropyl Methylcellulose Ophthalmic Solution, Appasamy Ocular Devices Private Limited, Solan, HP) were injected to maintain the anterior chamber. Loaded phakic IOL was injected slowly and steadily into the anterior chamber without damaging the corneal endothelium and anterior lens capsule. With the help of an IPCL manipulator, all haptics were tucked under the iris through the side port. Once haptics was placed in position, the viscoelastic was removed by using an irrigation and aspiration cannula. A digital image-guided system (Verion Image Guided System; Alcon, Fort Worth, TX, USA) enabled the measurement of intraoperative cyclotorsion and the correct placement of the TIPCL. Intracameral moxifloxacin was injected, and wounds were hydrated at the end. Postoperative treatment regimen included antibiotic drops (moxifloxacin ophthalmic solution 0.5%) for 1 week and steroids (loteprednol eye drops 0.5%) in tapering doses over 4 weeks, along with preservative-free lubricants. Postoperatively, all patients were reviewed on day 1, 1 week, 6 weeks, 6 months, and annually thereafter.

### Statistical Analysis

IBM SPSS version 24 was used for statistical analysis. Snellen visual acuity measurements were converted to logMAR equivalents for data analysis and interpretation. The continuous variables were presented with their means and standard deviations. The categorical variables were explained in terms of frequency and percentage. One-way ANOVA, repeated measures ANOVA, and an Independent t-test were used for the statistical analysis. The p-value ≤0.05 was considered statistically significant.

## Results

### Characteristics of the study participants

We included data from 86 eyes of 64 patients aged 30.67 ± 7.96 years with Male: Female ratio of 40:24. They were divided into three categories as CXL (Group1), INTACS combined with CXL (Group 2), and Topography-guided photorefractive keratectomy combined with CXL (group 3) based on their prior treatment for keratoconus and allowing for a detailed analysis of the outcomes across the different treatment approaches. The mean age was 30.67 ± 7.96, with a one-year follow-up period. Forty-two eyes underwent unilateral implantation, and 22 eyes had bilateral implantation. Preoperative demographics of the study population are summarized in **Table 1**.

**Table 1:** Participants demographic profile

Total number of patients	64
Total number of eyes	86
Group 1 (CXL)	77
Group 2 (INTACS+CXL)	6
Group 3 (TGPRK+CXL)	3
Age (mean ± SD)	30.67± 7.96
Sex (Male: Female)	40:24
Unilateral implantation	42
Bilateral implantation	22
Average follow-up period	One year

### Refractive outcome

**Fig. 1:**
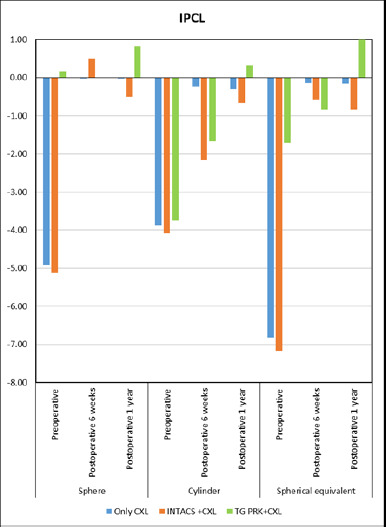
Graphical representation showing the subgroup analysis of spherical equivalent, sphere, and cylinder pre- and post-toric IPCL implantation in stabilized keratoconus patients

The mean spherical equivalent in the CXL group of TIPCL - 6.82 ± 4.63 diopters (D). In the INTACS+CXL group, the mean value was -7.17 ± 4.10 D. In the TG PRK+CXL group, it was -1.71 ± 0.83 D. One year postoperatively, the spherical equivalent in the CXL group was -0.15 ± 0.67 D, in the INTACS group, it was -0.83 ± 1.70 D (p < 0.001), and in the TGPRK group it was 1.00 ± 1.73 D. Similarly, preoperative spherical refractive power was -4.91 ± 4.82 D, -5.13 ± 4.78 D, 0.17 ± 1.26 D in group 1, 2 and 3. After one year, it was -0.02 ± 0.53 D, -0.50 ± 1.22 D, and 0.83 ± 1.44 D. Preoperative cylinder power changes from -3.87 ± 1.63 D, -4.08 ± 2.00 D, and -3.75 ± 1.30 D to -0.30 ± 0.78 D, -0.67 ± 1.08 D and 0.33 ± 0.58 D among three groups at one year follow up period (p < 0.001). The mean and standard deviation values for the sphere, cylinder, and spherical equivalent across the three groups are shown in **Table 2** and **Fig. 1**.

**Table 2:** Analysis of the changes in spherical equivalent, sphere, and cylinder from preoperative to postoperative across subgroups

Parameters	T IPCL			P value
	Group 1 (CXL only)	Group 2 (INTACS+ CXL)	Group 3 (TG PRK+CXL)	
1. Spherical Equivalent (mean ± SD)				
A. Preoperative	-6.82 ± 4.63	-7.17 ± 4.10	-1.71 ± 0.83	0.161
B. 6 weeks postoperative	-0.14 ± 0.33	-0.58 ± 1.88	-0.83 ± 1.44	0.043
C. 1 year postoperative	-0.15 ± 0.67	-0.83 ± 1.70	1.00 ± 1.73	0.008
P value	<0.001	<0.001	<0.001	
2. Sphere (mean ± SD)				
A. Preoperative	-4.91 ± 4.82	-5.13 ± 4.78	0.17 ± 1.26	0.196
B. 6 weeks postoperative	0.00 ± 0.31	0.50 ± 2.26	0.00 ± 0.00	0.174
C. 1 year postoperative	-0.02 ± 0.53	-0.50 ± 1.22	0.83 ± 1.44	0.015
P value	<0.001	<0.001	<0.001	
3. Cylinder (mean ± SD)				0.945
A. Preoperative	-3.87 ± 1.63	-4.08 ± 2.00	-3.75 ±1.30	0.174
B. 6 weeks postoperative	-0.23 ± 0.69	-2.17 ± 2.04	-1.67 ± 2.89	0.212
C. 1 year postoperative	-0.30 ± 0.78	-0.67 ± 1.08	0.33 ± 0.58	
P value	<0.001	<0.001	<0.001	

### Visual acuity

The mean UDVA in the CXL, INTACS, and TGPRK groups was logMAR 1.12 ± 0.58, 0.75 ± 0.23, and 0.57 ± 0.06, respectively. At one year, mean UCVA across the three groups increased to logMAR 0.17 ± 0.16, 0.28 ± 0.27, and 0.40 ± 0.53 for the CXL, INTACS, and TGPRK groups, respectively. The mean preoperative BCVA in the CXL, INTACS, and TGPRK groups was log MAR 0.15 ± 0.13, 0.23± 0.15, and 0.20 ± 0.10, respectively. The mean postoperative BCVA in the 1-year follow-up across the three groups was log MAR 0.11 ± 0.11, 0.23 ± 0.25, and 0.18 ± 0.10, respectively, and this difference was statistically significant (p < 0.001) in the CXL group. The UDVA and BDVA changes are depicted in **Table 3**. The uncorrected and best-corrected distance visual acuity improved significantly in the postoperative visits compared with the preoperative UDVA and BDVA. The results were statistically significant in each subgroup, except in the TGPRK+CXL subgroup, which was attributed to the subgroup’s small sample size.

**Table 3:** Comparison of pre- and postoperative UDVA and BDVA in patients undergoing TIPCL implantation following CXL, INTACS, and TGPRK

	T IPCL			P value
UCVA Log Mar (mean ± SD)	Group 1 (CXL only)	Group 2 (INTACS+(INTACS+CXL)	Group 3(TG PRK+CXL)	
		0.75 ± 0.23	0.57 ± 0.06	
A. Preoperative	0.68± 0.58	0.35 ± 0.23	0.57 ± 0.06	0.086
B. 6 weeks postoperative	0.18 ± 0.17	0.28 ± 0.27	0.40 ± 0.53	0.031
C. 1 year postoperative	0.17 ± 0.16	<0.001	<0.001	0.054
P value	<0.001			
BCVA Log Mar (mean ± SD)	0.15 ± 0.13	0.23± 0.15	0.20 ± 0.10	0.255
A. Preoperative 6 weeks postoperative	0.14 ± 0.15	0.22 ± 0.04	0.23 ± 0.15	0.222
B. 1 year postoperative	0.11 ± 0.11	0.18 ± 0.10	0.23 ± 0.25	0.086
P value	<0.001	0.002	0.135	

### Safety and efficacy outcomes

**Fig. 2:**
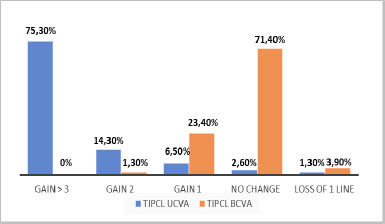
The gain and loss of lines in UDVA and BDVA by comparing the results before and after Toric IPCL surgery in keratoconus patients

**Fig. 3:**
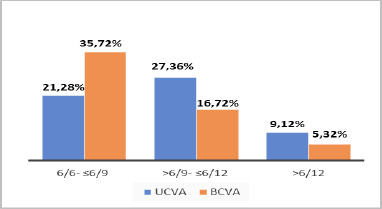
Cumulative Snellen Visual Acuity improvements after Toric implantable phakic contact lens in stable keratoconus cases

At the 1-year postoperative visit, 75.3% gained more than three lines of UDVA, and 71.4% maintained the same BDVA. At the 1-year postoperative visit, 65.36% attained best-corrected distance visual acuity (BDVA) of 20/20 to 20/32, as shown in **Fig. 3. Fig. 2** highlights the change in UDVA and CDVA. **Fig. 3** shows the cumulative Snellen visual acuity. The average safety index was 0.77, and the efficacy index was 1.20 at the end of 1-year follow-up (**Table 4**).

**Table 4:** One-year efficacy and safety index analysis in distinct subgroups

Groups	Efficacy index	Safety index
Group 1 (Only CXL)	0.155	0.748
Group 2 (INTACS+CXL)	0.378	0.786
Group 3 (TG PRK+CXL)	0.706	1.167
In General	1.207	0.770

### Adverse events

Rotation of the posterior chamber toric phakic IPCL was performed in 4 cases to correct misalignment within ten days of implantation. On postoperative day 1, two patients exhibited a shallow anterior chamber with a positive Seidel’s sign at the incision wound. AC reformation was carried out on the same day for both patients. Significant corneal edema was noted in two patients, one of whom also had a small peripheral Descemet’s membrane detachment at the main incision. Both patients experienced incorrect (flipped) TIPCL implantation during insertion, necessitating the removal and reinsertion of the lenses. One patient, with an older TIPCL model lacking central holes, required an Nd: YAG laser peripheral iridotomy after one year, due to high vault and progressive anterior chamber shallowness. One patient experienced progression of keratoconus during follow-up, with K-max increasing from 45.0D to 47.0D and a reduction of 35 microns in corneal thickness, and underwent repeat CXL. One patient experienced a grade 1 corneal haze after CXL, which persisted after TIPCL implantation. Additionally, an anterior subcapsular cataract was identified in the midperipheral part of the crystalline lens in one patient during the follow-up period. All the complication rates are shown in **Table 5**.

**Table 5:** Summary of intraoperative and postoperative complications

Complications	Number of patients (86)
Intraoperative	
1. Flipped Phakic IOL implantation	2 (1.72%)
Postoperative	
1. Phakic IOL Rotation (due to misalignment)	4 (3.44%)
2. Shallow anterior chamber (due to wound leak)	2 (1.72%)
3. Progressive Shallow anterior chamber (due to high vault)	1 (0.86%)
4. Progression of Keratoconus	1 (0.86%)
5. Anterior subcapsular cataract	1 (0.86%)

## Discussion

This retrospective interventional cross-sectional study, conducted on 86 keratoconus eyes, primarily focuses on evaluating the efficacy and safety of toric phakic IPCL implantation followed by various treatments (CXL, INTACS, TGPRK) to correct myopia and astigmatism across different ranges, after refractive stabilization with a one-year follow-up period. After an extensive review of the literature, our study is the first to compare the outcomes of toric phakic IPCL implantation following CXL, INTACS, and TGPRK to correct residual refractive error.

Collagen crosslinking is a crucial step in reinforcing corneal biomechanics and serves as a benchmark for multiple interventions aimed at addressing the refractive component of the disease. Caporossi et al. reported a decrease in K readings by more than 2.0D, a mean postoperative SE exceeding 1.0D, and a reduction of 1.0D in cylinder. Additionally, it has a synergistic effect in lowering corneal wavefront aberrations following CXL [11]. To achieve the best possible visual outcome in keratoconus patients, in terms of both visual acuity and quality, remains a challenging undertaking for refractive surgeons, even after inhibiting progression with collagen cross-linking, ICRS, and topography-guided laser treatment integrated with CXL, which minimizes irregularity and has limited efficacy in correcting spherocylindrical errors. It is tailored depending on the baseline refraction, corrected distance visual acuity, corneal irregularity, astigmatism, and the stage of the disease.

Phakic IOL is chosen for patients who have substantial residual refractive errors with or without anisometropia and have satisfactory spectacle vision following CXL. Lens power and size are assigned by the manufacturer based on refined refraction, k readings, ACD, pachymetry, WTW, and axial length. WTW measurement is taken both digitally and manually. The target was to achieve complete correction of the remaining sphere and cylinder.

After one year, this study showed that 54.18% achieved a UDVA of 20/20 to 20/32, and 65.36% attained a BDVA of 20/20 to 20/32. Mean SE was 0, 0.15, 0.83, and 1.00 (p value <0.001). Spherical and astigmatism were significantly reduced, with a p-value of <0.001. 75.3% gained more than three UDVA lines, and 71.4% maintained the same BDVA at the end of one year. The safety and efficacy indices at 1 year were 0.770 and 1.207. In terms of UDVA and BDVA gains, the results of this study are comparable to other reports, as evidenced by the safety and efficacy indices.

Shaheen S et al. study demonstrated an improvement in mean preoperative BDVA from 0.56 ± 0.13 to 0.89 ± 0.17 (p=0.0001), and mean UDVA enhanced from 0.63 ± 0.14 to 0.88 ± 0.18 after three years in 16 eyes with early keratoconus. The safety and efficacy indices were -0.12 ± 0.09 and -0.06 ± 0.11, respectively [12]. Seyed J et al. analyzed the five-year outcomes of toric and non-toric phakic IOL in 23 keratoconus eyes of 13 patients. Mean preoperative spherical equivalent and cylinder changed from 5.35 ± 2.82 D to 1.56 ± 1.53 D. Mean UDVA and BDVA changed to 0.74 ± 0.22 and 0.88 ± 0.16. Mean safety and efficacy indices were 1.47 ± 0.32 and 1.24 ± 0.34. Nineteen eyes gained one or more lines, and none showed loss of lines [13]. A case report by Kymionis et al. demonstrated good outcomes following the implantation of phakic IOLs after one year of CXL. UDVA improved from counting fingers to 20/40, and BDVA improved from 20/100 to 20/30 at 3 months post procedure [14]. Izquierdo et al. and Guell et al. published the outcomes of foldable iris-claw phakic IOL. All eyes reached the final spherical and cylindrical error, ranging from 0 to -1.50 D and 0 to -1.75 D, and the second study reported that the postoperative UDVA was 20/40 or better in 16 eyes (94%). Fourteen eyes (82%) were within ± 0.50 diopter (D) of the attempted SE correction, and 13 eyes (76%) were within ± 1.00 D of the attempted cylinder correction [15,16]. Few studies have investigated the implantation of phakic intraocular lenses in patients with keratoconus, specifically without performing corneal crosslinking (CXL). Notable studies include those by Alfonso et al., Kamiya et al., and Hashemian et al., which all focused on early-stage keratoconus cases. These studies reported favorable outcomes in terms of safety and predictive results at the 1-year postoperative period [17-19].

The outcomes of our study are consistent with those reported in the referenced articles. However, direct comparison with other studies is challenging due to variations in case numbers and the fact that only post-CXL patients were selected in the reviewed studies.

The most prevalent complications after phakic IOL implantation included elevated IOP and anterior subcapsular cataracts, with the most common causes for explantation being improper sizing, followed by cataract development [20]. Other rare complications like chronic persistent increased IOP-induced disc damage, retinal detachment, toxic anterior segment syndrome, reverse implantation, and Descemet’s membrane damage have also been reported [21,22]. None of these complications were encountered, except in one patient who showed an anterior subcapsular cataract in the midperipheral region, which caused a decline in postoperative BDVA to 6/18 from preoperative BDVA of 6/12. This patient was closely monitored for progression over 3 years postoperatively and maintained the same vision.

One of the most critical factors that guarantees the success of implanting Phakic IOLs in patients with keratoconus is the adequate selection of patients. It is the personal protocol of the main author of the present study to consider the implantation of TIPCL in patients who achieve a BDVA of 20/40 or better. The above is justified by the fact that, being in the intraocular space, phakic IOL does not cause any corneal aberrations, so the vision after their implantation will be much more similar to that obtained with glasses than that obtained with contact lenses.

## Conclusion

Implantation of phakic IOLs is a valuable option for visual rehabilitation in keratoconus patients. The use of toric IPCL has been proven safe and effective, resulting in visual improvement for all participants in our study. However, further randomized, comparative, and long-term studies with larger sample sizes are needed to assess the durability of the procedure and to identify the best treatment methods.

This study is limited by its retrospective approach and the small cohort in the topography-guided photo-refractive keratectomy group. To mitigate statistical bias, a one-year postoperative follow-up period was established; however, some patients were monitored for a longer duration, ranging from three to five years.
